# Optimization of Ultrasonic-Assisted Extraction of Diene Urushiol from Lacquer Tree Leaves Using Response Surface Methodology

**DOI:** 10.3390/molecules30081663

**Published:** 2025-04-08

**Authors:** Fengming Xia, Haojiang He, Jize Ma, Yutian Jin, Qing Qiao, Peng Long, Ping Li, Rui Sun

**Affiliations:** 1 College of Biological Science and Food Engineering, Southwest Forestry University, Kunming 650224, China; 16687002536@163.com (F.X.); hehj01@swfu.edu.cn (H.H.); Xanny73@163.com (J.M.); jinyutian2025@163.com (Y.J.); 15291762523@163.com (Q.Q.); lp18313786420@163.com (P.L.); 2 Key Laboratory for Forest Resources Conservation and Utilization in the Southwest Mountains of China, Ministry of Education, Southwest Forestry University, Kunming 650224, China; liping2020@swfu.edu.cn

**Keywords:** lacquer tree, diene urushiol, ultrasound-assisted extraction, response surface methodology (RSM)

## Abstract

Lacquer trees are an important economic tree species in China, and raw lacquer is its main secondary metabolite. Polyphenolic compounds are the primary components of raw lacquer, among which diene urushiol exhibits high inhibitory activity against the reverse transcriptase of acquired immunodeficiency syndrome (AIDS). Therefore, this study established and optimized the ultrasound-assisted extraction process of diene urushiol from lacquer tree leaves. Based on single-factor experiments on the number of extractions, extraction time, extraction temperature, and solvent to solid ratio, the Box–Behnken Design response surface methodology was employed to obtain the optimal extraction process, which included three extractions, an extraction time of 55 min, an extraction temperature of 50 °C, and a solvent to solid ratio of 10:1 mL/g. Under these conditions, the content of diene urushiol was 4.56 mg/g (FW), which bore no significant difference from the theoretical value of 4.69 mg/g (FW), indicating a good model fit. Therefore, response surface methodology (RSM) can be used to optimize the extraction process of diene urushiol from lacquer leaves. This method lays a solid foundation for the comprehensive development and utilization of lacquer tree resources.

## 1. Introduction

Lacquer trees, *Toxicodendron vernicifluum* (Stokes) F.A. Barkley, are deciduous trees belonging to the *Anacardiaceae* family and the *Toxicodendron* genus and are important, special economic trees in China [1]. Raw lacquer is the main secondary metabolite of lacquer trees and is regarded as a natural resin coating, often referred to as the “king of coatings”. Urushiol is the primary component (comprising 40% to 70%) in raw lacquer and mainly includes saturated monoene, diene, and triene urushiols with C15 or C17 [2]. Urushiol possesses antioxidant [3], antitumor [4] antibacterial [5], and immune-regulating [6] properties. Studies have reported that diene urushiol exhibits high inhibitory activity against the reverse transcriptase of acquired immunodeficiency syndrome (AIDS), which can help prevent and treat AIDS [7].

Surveys indicate that over half of the individuals exposed to lacquer trees develop contact dermatitis [8]. Due to this particularity, the development and utilization of lacquer trees are limited, leading to their nickname, “toxic lacquer tree”. Currently, the primary use of lacquer trees involves harvesting raw lacquer from the bark for coatings and extracting oil from their seeds [9]. Research indicates that lacquer tree leaves contain urushiol similar to those found in raw lacquer [2]. Consequently, lacquer tree leaves, being a renewable resource, can serve as raw materials for extracting urushiol for efficient utilization of lacquer tree plant resources.

Currently, the extraction methods mainly include solvent extraction via thermal reflux [10,11], ultrasound-assisted extraction [12], microwave-assisted extraction [13], and aqueous two-phase extraction [14]. However, thermal reflux has a long extraction time, microwave-assisted extraction has high energy consumption, and aqueous two-phase extraction consumes solvent because of complex phase separation [15]. Ultrasound-assisted extraction is more commonly used for extracting phenolic compounds because it employs the combined effects of cavitation, vibration, crushing, and stirring generated by ultrasound waves to disrupt cell walls, achieving efficient extraction of cellular contents. Therefore, ultrasound-assisted extraction can effectively solve the problems of long extraction time, high energy consumption, and low yield. However, there have been no reports on the ultrasound-assisted extraction of urushiol from lacquer tree leaves. When bioactive components are extracted from plants by ultrasonic-assisted extraction, the factors that can influence the extraction include extraction temperature, extraction time, the number of extractions, the solvent to solid ratio, etc. [16,17,18]. To attain optimum extraction efficiency, we conducted research on these parameters.

**Figure 1:**
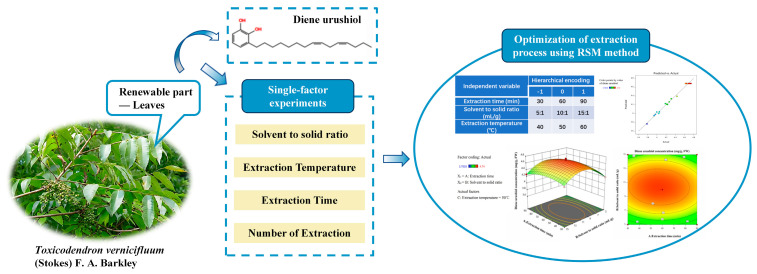
The experimental scheme of this study.

Response surface methodology (RSM) is a method that involves experimental design, model establishment, and data analysis to evaluate the interactions between factors across a region and to determine optimal conditions and response values [18,19]. It is commonly used in the extraction studies of plant components [20]. Therefore, this study used the renewable part of lacquer tree leaves as raw materials and optimized the ultrasound-assisted extraction process of diene urushiol using the RSM method, based on single-factor experiments (Figure 1). The purpose of this study is to improve the extraction efficiency of diene urushiol and make full use of lacquer tree plant resources. Ultimately, it lays the foundation for the comprehensive utilization of lacquer trees.

## 2. Results and Discussion

### 2.1. High-Performance Liquid Chromatography (HPLC) Analysis

HPLC analysis was conducted using the concentration of the diene urushiol standard as the x-axis (X, mg/mL) and the peak area as the y-axis (Y), resulting in a linear regression equation for the standard curve of diene urushiol:

y = 3590x − 27.327 (R^2^ = 0.993) (1)

**Figure 2:**
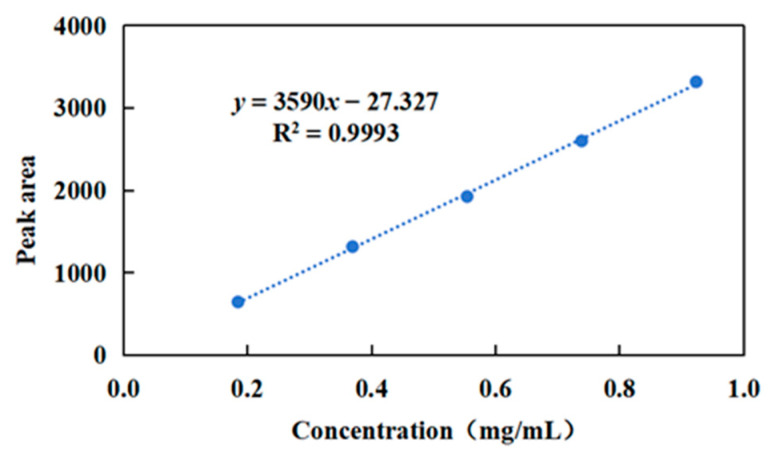
Standard curve of diene urushiol.

A good linear relationship was observed within the range of 0.1847 to 0.9235 mg/mL (Figure 2).

### 2.2. Results of Single-Factor Experiments

#### 2.2.1. Effect of Solvent to Solid Ratio on the Extraction Content of Diene Urushiol

**Figure 3:**
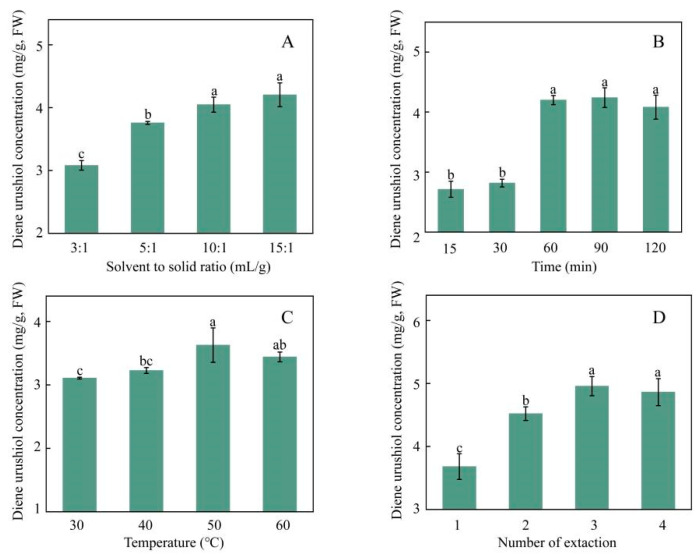
Effects of solvent to solid ratio (**A**), extraction time (**B**), extraction temperature (**C**), and number of extractions (**D**) on the content of diene urushiol. Different letters indicate the statistical significance at *p* < 0.05.

Increasing the solvent to solid ratio can enhance the solubility of the target components in the solvent, and the amount of extraction solvent significantly affects the extraction of the target components [21,22,23]. Sofi et al. reported optimal solvent to solid ratio of 8:1 mL/g for total phenolic compounds recovery from olive pomace oil [24]. A minimum solvent to solid ratio of 3:1 mL/g was found to ensure complete immersion of the sample powder, whereas a maximum ratio of 15:1 mL/g, which is approximately double that reported by Sofi et al., was sufficient for the extraction of diene urushiol. Therefore, in this study, the effects of four different solvent to solid ratios of 3:1, 5:1, 10:1 and 15:1 mL/g. The effect of the solvent to solid ratio on the content of diene urushiol was investigated, and the results are shown in Figure 3A. The results indicate that as the solvent to solid ratio increases from 3:1 to 10:1 mL/g, the content of diene urushiol significantly increases; however, when the ratio is further increased from 10:1 to 15:1 mL/g, the content of diene urushiol no longer shows significant changes. This suggests that at a solvent to solid ratio of 10:1 mL/g, diene urushiol is effectively extracted in the solvent. Chen et al. investigated ultrasonic-assisted extraction of flavonoids, polysaccharides, and eleutherosides from Acanthopanax senticosu, and the optimal solvent to solid ratio was 10:1 mL/g [25]. This result is similar to our study. Therefore, the solvent to solid ratio range of 5:1 to 15:1 mL/g was selected in the further RSM studies.

#### 2.2.2. Effect of Extraction Time on the Extraction Content of Diene Urushiol

Increasing extraction time is beneficial for the extraction of effective components. However, it also increases the cost of time and energy [26]. Rosa et al. identified an optimal extraction time of 113.6 min within a 20–120 min range for total phenolic compounds extraction from grape peels [27]. On this basis, the present study investigated the effects of five different extraction times on the content of diene urushiol was investigated, and the results are shown in Figure 3B. From 15 to 30 min, the content of diene urushiol did not change with increasing time; however, from 30 to 60 min, there was a significant increase in the content of diene urushiol. After 60 min, the content no longer changed. This indicates that 60 min is sufficient for the complete extraction of diene urushiol. Therefore, the extraction time range of 30 to 90 min was selected in the following study.

#### 2.2.3. Effect of Extraction Temperature on the Extraction Content of Diene Urushiol

Temperature has a significant impact on extraction performance, and an increase in ultrasonic temperature may lead to a higher diffusion coefficient of the target compounds and enhance their solubility [28]. Liao et al. determined an optimal temperature of 44.5 °C for total phenolic compounds extraction from purple eggplant peels [29]. Based on this, the present study investigated the effects of four different extraction temperatures of 30, 40, 50 and 60 °C on the content of diene urushiol, and the results are shown in Figure 3C. The content of diene urushiol significantly increased with the rise in extraction temperature from 30 to 50 °C. However, when the extraction temperature reached 50 °C, further increasing the temperature resulted in a decrease in the content of diene urushiol, possibly due to degradation of the compound at higher temperatures [30]. Diene urushiol degrades above 50 °C, which is similar to the thermal stability trends of curcumin and anthocyanins [29]. Therefore, it is believed that 50 °C is a suitable temperature for extracting diene urushiol. The extraction temperature range of 40 to 60 °C was selected in the following study. Although diene urushiol exhibits good bioactivity, its stability under long-term storage conditions still needs further study [31].

#### 2.2.4. Effect of the Number of Extractions on the Extraction Content of Diene Urushiol

Before the active components are extracted completely, the content of active components can increase by increasing the number of extractions. However, as the number of extractions increases, more solvent is used, and more time is consumed [32]. Therefore, selecting the appropriate number of extractions is crucial. Wang et al. investigated the effect of the number of extractions on phenolic compound yields from Inula helenium within the number range of 1–4 [33]. Based on this, in the present study the effects of four different numbers of extractions at 1, 2, 3, and 4 on the diene urushiol content were studied, and the results are shown in Figure 3D. The content of diene urushiol significantly increased from extractions one to three. After the third extraction, the content of diene urushiol no longer changed, indicating that it was fully extracted at this point. Therefore, three extractions is energy-efficient and preferable.

### 2.3. Establishment of the Response Surface Box–Behnken Design

RSM design typically focuses on studying the interactions between continuous variable factors, while the number of extractions is often a discrete integer variable. Its variation lacks continuity, which may make it less suitable for fitting response surface models. The factor of extraction frequency was not included in the subsequent response surface optimization research. The effect of extraction frequency was only studied in single-factor experiments. Therefore, solvent to solid ratio, extraction time, and extraction temperature were selected for further optimization of the response surface model. In the Box–Behnken design (BBD) method of response surface methodology, the true values and coded levels of the independent variables are shown in Table 1. Seventeen experimental groups were obtained through response surface experimental design, and the results are shown in Table 2.

### 2.4. Model Fitting Statistics and Variance Analysis

Design Expert 11.0 software was used for analysis, and the quadratic multiple regression equation for the extraction content of diene urushiol (Y) is

Y = 4.68 − 0.0199A + 0.0456B + 0.0626C − 0.016AB − 0.1566AC − 0.0722BC − 0.1101A^2^ − 0.3166B^2^ + 0.4192C^2^ (R^2^ = 0.9876) (2)

Among them, Y represents the extraction concentration of the diene urushiol, A represents extraction time (min), B represents solvent to solid ratio (mL/g), and C represents extraction temperature (°C).

**Figure 4:**
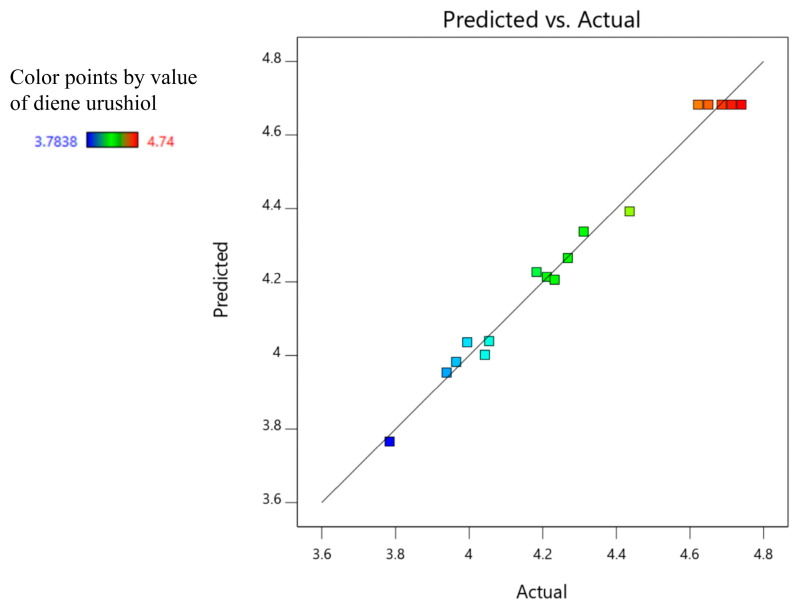
Predicted value and actual value of the contents for diene urushiol.

The analysis of variance (ANOVA) was conducted for each factor related to the content of diene urushiol. In Table 3, it can be seen that the model is highly significant (*p* < 0.0001, f = 62.17), and the lack of fit is not significant (*p* > 0.05, f = 1.44). It can be observed that the linear terms of the solvent to solid ratio and extraction temperature have a significant effect on the diene urushiol content (*p* < 0.05). The two-way interaction between extraction time and extraction temperature (*p* < 0.01) has a greater impact on the diene urushiol content than that between solvent to solid ratio and extraction temperature (*p* < 0.05). When the interaction of extraction time and temperature is significant (*p* < 0.01), prolonging the extraction time at a high temperature will exacerbate the degradation phenomenon [34], which is similar to the result found in this study. The quadratic terms of the solvent to solid ratio and extraction temperature have an extremely significant effect on the diene urushiol content (*p* < 0.0001), and the quadratic term of the extraction time has a highly significant impact on the diene urushiol content (*p* < 0.01). A higher statistical correlation coefficient (R^2^ > 0.90) indicates a good fit between experimental values and those obtained by the models [35]. The statistical correlation coefficient R^2^ is 0.9876 in this study, which is an ideal observation (R^2^ > 0.90) for the fitted model, proving that the experimental values fit well with the model values. The distribution of actual values approximates a straight line, further confirming the model’s adequacy for predicting and analyzing experimental results in Figure 4.

### 2.5. Analysis of Response Surface Interactions

**Figure 5:**
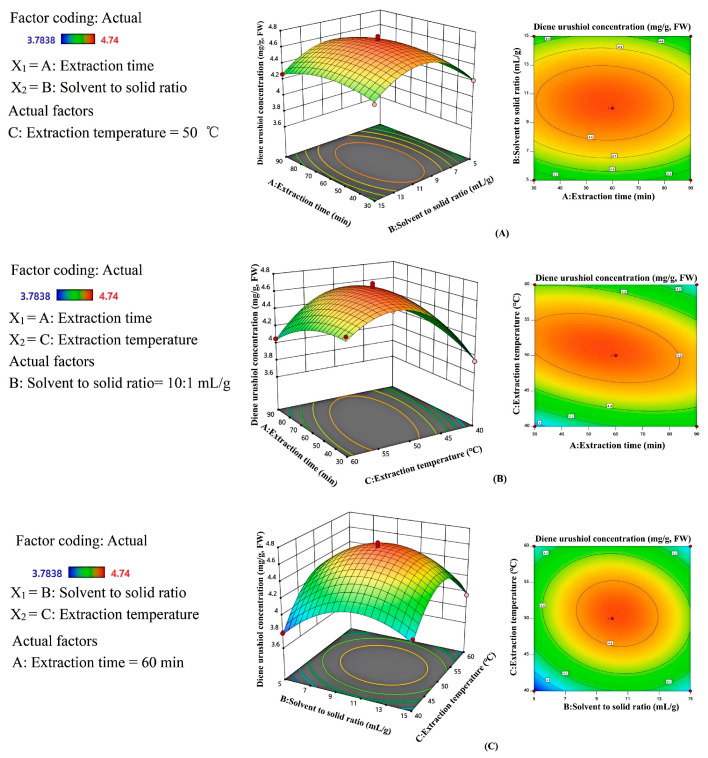
Interaction of different variables in the response surface. (**A**) extraction time and solvent to solid ratio, (**B**) extraction time and extraction temperature, (**C**) solvent to solid ratio and extraction temperature.

The three-dimensional response surface plot can visually indicate the impact of each variable at different experimental levels and the type of interaction between two variables to determine the optimal conditions for maximizing the response value [36]. Steep curves in the three-dimensional response surface plot indicate significant interactions between the factors, while flat curves suggest that the effects of these factors are not significant [18,19,35]. In Figure 5A, it can be observed that when the solvent to solid ratio is constant, the 3D surface for the extraction time is not steep, indicating that the interaction between solvent to solid ratio and extraction time is not significant. Figure 5B,C show a peak in the 3D surface, with isopleths appearing elliptical. This indicates that the interaction between extraction time and extraction temperature, as well as the interaction between solvent to solid ratio and extraction temperature, has a significant impact on the content of diene urushiol. In Figure 5B, it can be observed that when the temperature varies from 40 to 60 °C, the content of diene urushiol increases and then decreases. This may be due to the increase in molecular diffusion rate with the rising temperature at lower temperatures, but further temperature increases can negatively affect the bioactive substances [37], leading to a decrease in the extraction content of diene urushiol. Additionally, it can be seen from Figure 5C that when the extraction temperature and/or the solvent to solid ratio are low or high, the extraction content of diene urushiol is low. A higher extraction amount of diene urushiol can be obtained only with moderate extraction temperature and solvent to solid ratio. At the same time, it can be observed that the surface steepness on the extraction temperature side is greater than that on the solvent to solid ratio side. This indicates that under the influence of their interaction, the extraction temperature has a greater impact on the extraction content of diene urushiol than the solvent to solid ratio. The results of the above analysis are consistent with the results of the variance analysis.

### 2.6. Model Validation

**Figure 6:**
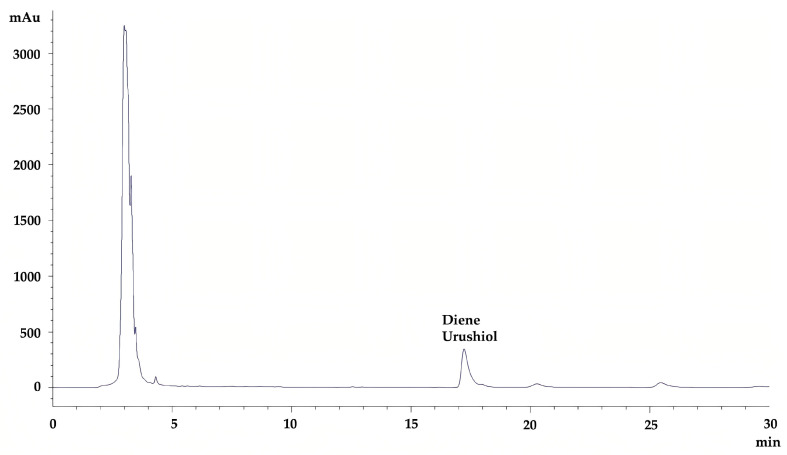
The chromatogram of the sample solution.

Through Design Expert 11.0 software, the optimal conditions predicted by the model were obtained as follows: extraction time of 55 min, extraction temperature of 51 °C, and solvent to solid ratio of 10.3:1 mL/g. Under these conditions, the predicted content of diene urushiol was 4.69 mg/g, FW. Considering the feasibility of practical operations, the extraction process for diene urushiol was adjusted to an extraction time of 55 min, extraction temperature of 50 °C, and solvent to solid ratio of 10:1 mL/g. Under this extraction process, the content of diene urushiol obtained was 4.56 mg/g, FW, which bore no significant difference compared to the model-predicted value of 4.69 mg/g, FW (RSD of 2.26%). The chromatogram of the sample solution obtained under optimal extraction conditions is presented in Figure 6.

## 3. Materials and Methods

### 3.1. Materials and Reagents

The lacquer tree leaves were collected from wild lacquer trees in Dachongzi Village in August 2022, Wenshan City and Miao Autonomous Prefecture, Yunnan Province of China. After harvesting, they were immediately stored in a −80 °C freezer and ground into powder using liquid nitrogen during the experiment. The standard of diene urushiol with 98% purity was purchased from Shanghai Dande Biotechnology Co., Ltd. (Shanghai, China). Analytical-grade ethanol was purchased from Guangdong Guanghua Technology Co., Ltd. (Shantou, China). Chromatographic acetonitrile was purchased from Beijing Meiliade Technology Co., Ltd. (Beijing, China), and ultrapure water was made by a water purifier (PP010XXM1, ELGA, Warwickshire, UK) for all experiments.

### 3.2. Quantitative Determination of Diene Urushiol by HPLC

The Agilent high-performance liquid chromatography system (Agilent Technologies, Santa Clara, CA, USA) was used for the determination of diene urushiol, comprising an automatic injector, a quaternary pump, a DAD UV detector, and an EC-C18 column (250 mm × 4.6 mm; 4 µm). The mobile phase consisted of acetonitrile and water (83:17), with a flow rate of 0.7 mL/min, and the analytical time for a single sample was 30 min. The column temperature was maintained at 40 °C, the detection wavelength was set at 225 nm, and the injection volume was 2 µL. A precise amount of diene urushiol standard was accurately weighed and gradient-diluted using chromatographic methanol (Beijing Bailingwei Technology Co., Ltd., Beijing, China) to obtain the standard solution of varying concentrations (0.1847, 0.3694, 0.5541, 0.7388 and 0.9235 mg/mL). According to the HPLC procedure, the peak areas corresponding to different concentrations of the standard solutions were detected, and a standard curve was established to show the linear relationship between peak area and diene urushiol content.

### 3.3. Single-Factor Experimental Design

100% ethanol was used as the extraction solvent, and ultrasonic-assisted extraction was employed to extract diene urushiol from lacquer tree leaves under specific conditions of solvent to solid ratio, extraction time, extraction temperature, and extraction frequency. Fresh lacquer tree leaves were ground into powder using liquid nitrogen, and 1.00 g was weighed and placed in a centrifuge tube. Corresponding volumes of anhydrous ethanol solution were added according to specific solvent to solid ratios (3:1, 5:1, 10:1, and 15:1 mL/g). The mixture was subjected to ultrasonic extraction at specific temperatures (30, 40, 50, and 60 °C) in an ultrasonic bath (SK5210HP, Shanghai Kudos Ultrasonic Instrument Co., Ltd., Shanghai, China) for specified durations (15, 30, 60, 90, and 120 min). It was then centrifuged at 12,000 rpm for 10 min in a centrifuge (R134a, Eppendorf AG, Hamburg, Germany), and the supernatant was collected for use. The extraction was conducted with a certain frequency (1, 2, 3, and 4 times). All the supernatants were mixed, then concentrated to dryness by using rotary evaporation. Then, the residue was dissolved in 2 mL of anhydrous ethanol solution for HPLC analysis. All sample solutions were filtered through a membrane with a pore size of 0.22 µm before conducting HPLC analysis. Each treatment was repeated three times.

### 3.4. Response Surface Design

The Box–Behnken design (BBD), an important experimental design method in RSM, is widely used in scientific research and industrial production optimization due to its efficiency and accuracy [31]. Based on single-factor experiments, extraction time (A), solvent to solid ratio (B), and extraction temperature (C) were identified as three significant factors affecting the content of diene urushiol. Therefore, a response surface methodology (RSM-BBD) experimental design was conducted using these three factors at three levels. The extraction content of diene urushiol was set as the response value, and a second-order polynomial model was established to determine the regression coefficients. The experiments conducted based on the RSM experimental design, the diene urushiol contents were determined, and variance analysis was conducted to test the significance of the model concerning the dependent variable (*p* < 0.05). Three-dimensional response surface curves were also plotted to analyze the interaction effects of the factors on the response value. Finally, the optimal ultrasonic extraction process parameters were determined and validated by the response surface prediction model.

### 3.5. Statistical Analysis

Statistical analysis of experimental data was performed using Excel software (2019, Microsoft Office, Redmond, WA, USA), followed by Duncan’s multiple range test and one-way ANOVA using IBM SPSS Statistics 26.0 (Statistical Product Service Solutions, Armonk, NY, USA). Experimental design, ANOVA, modelling, and predictive optimization were carried out using Design Expert 11.0 software (Stat Ease, Inc., Minneapolis, MN, USA).

## 4. Conclusions

This study used lacquer tree leaves as experimental materials and optimized the effects of three extraction factors (solvent to solid ratio, extraction time, and extraction temperature) on the ultrasonic-assisted extraction of diene urushiol using the Box–Behnken Design method of response surface methodology. The results indicated that the optimal extraction conditions were as follows: extraction time of 55 min, extraction temperature of 50 °C, and solvent to solid ratio of 10:1 mL/g. Under these extraction conditions, the content of diene urushiol was 4.56 mg/g (FW), while the theoretical value was 4.69 mg/g (FW). They showed no significant difference, with an RSD of 2.82% and a good model fit. Therefore, this method can be used to optimize the ultrasonic-assisted extraction process of diene urushiol from lacquer tree leaves, providing an effective approach for the comprehensive and sustainable development and utilization of lacquer tree resources. In the future, scale-up and purified experiments can be carried out to obtain pure diene urushiol for pharmaceutical applications.

## Figures and Tables

**Table 1:** Independent variables of BBD experiment and their levels.

Independent Variable	Symbol	Hierarchical Encoding		
		−1	0	1
Extraction time (min)	A	30	60	90
Solvent to solid ratio (mL/g)	B	5:1	10:1	15:1
Extraction temperature (°C)	C	40	50	60

**Table 2:** Experimental design and results of response surface analysis.

Serial Number	Extraction Time (min)	Solvent to Solid Ratio (mL/g)	Extraction Temperature (°C)	Diene Urushiol Content ^1^ (mg/g, FW)
1	90	10:1	60	4.05
2	60	10:1	50	4.65
3	60	10:1	50	4.71
4	60	10:1	50	4.74
5	30	10:1	40	3.94
6	90	5:1	50	4.23
7	60	15:1	60	3.96
8	90	15:1	50	4.27
9	30	15:1	50	4.31
10	60	15:1	40	4.04
11	60	10:1	50	4.62
12	90	10:1	40	4.18
13	30	5:1	50	4.21
14	60	5:1	40	3.78
15	30	10:1	60	4.44
16	60	10:1	50	4.69
17	60	5:1	60	3.99

^1^ Each value is the mean of triplicate measure, n = 3.

**Table 3:** Analysis of variance of regression model.

Source	Sum of Squares	df	Mean Square	F-Value	*p*-Value
Model	1.4900	9	0.1658	62.1700	<0.0001 ***
A-Extraction time	0.0032	1	0.0032	1.1900	0.3110
B-Solvent to solid ratio	0.0166	1	0.0166	6.2400	0.0411 *
C-Extraction temperature	0.0314	1	0.0314	11.7700	0.0110 *
AB	0.0010	1	0.0010	0.3840	0.5551
AC	0.0981	1	0.0981	36.8000	0.0005 **
BC	0.0209	1	0.0209	7.8300	0.0266 *
A^2^	0.0511	1	0.0511	19.1500	0.0033 **
B^2^	0.4220	1	0.4220	158.2700	<0.0001 ***
C^2^	0.7400	1	0.7400	2.7751	<0.0001 ***
Residual	0.0187	7	0.0027		
Lack of fit	0.0097	3	0.0032	1.4400	0.3554
Pure error	0.0090	4	0.0022		
Cor total	1.5100	16			

* means significant at *p* ≤ 0.05; ** means highly significant at *p* ≤ 0.01; *** means extremely significant at *p* ≤ 0.0001.

## Data Availability

The data presented in this study are available in the article.
